# Progress in Electrohydrodynamic Atomization Preparation of Energetic Materials with Controlled Microstructures

**DOI:** 10.3390/molecules27072374

**Published:** 2022-04-06

**Authors:** Lihong Chen, Chengbo Ru, Hongguo Zhang, Yanchun Zhang, Hongxing Wang, Xiuli Hu, Gang Li

**Affiliations:** 1 Fire & Explosion Protection Laboratory, Northeastern University, Shenyang 110819, China; chenlihong@cipuc.edu.cn (L.C.); ligang@mail.neu.edu.cn (G.L.); 2 College of Forensic Science, Criminal Investigation Police University of China, Shenyang 110035, China; zhanghongguo@cipuc.edu.cn (H.Z.); zhangyanchun@cipuc.edu.cn (Y.Z.); 3 Key Laboratory of Impression Evidence Examination and Identification Technology, Ministry of Public Security, Shenyang 110035, China; 4 Graduate School, Shenyang Ligong University, Shenyang 110159, China; ljyywanghongxing@163.com; 5 School of Materials Engineering, Changshu Institute of Technology, Changshu 215500, China; mnhuxiuli@cslg.edu.cn

**Keywords:** electrospray, electrospinning, energetic materials, microstructure, reactivity

## Abstract

Constructing ingenious microstructures, such as core–shell, laminate, microcapsule and porous microstructures, is an efficient strategy for tuning the combustion behaviors and thermal stability of energetic materials (EMs). Electrohydrodynamic atomization (EHDA), which includes electrospray and electrospinning, is a facile and versatile technique that can be used to process bulk materials into particles, fibers, films and three-dimensional (3D) structures with nanoscale feature sizes. However, the application of EHDA in preparing EMs is still in its initial development. This review summarizes the progress of research on EMs prepared by EHDA over the last decade. The morphology and internal structure of the produced materials can be easily altered by varying the operation and precursor parameters. The prepared EMs composed of zero-dimensional (0D) particles, one-dimensional (1D) fibers and two-dimensional (2D) films possess precise microstructures with large surface areas, uniformly dispersed components and narrow size distributions and show superior energy release rates and combustion performances. We also explore the reasons why the fabrication of 3D EM structures by EHDA is still lacking. Finally, we discuss development challenges that impede this field from moving out of the laboratory and into practical application.

## 1. Introduction

Energetic materials (EMs), comprised of propellants, explosives and pyrotechnics, which can release abundant heat and considerable gaseous products within microseconds to milliseconds, are being widely used for both military and civilian purposes. Organic explosives are usually homogeneous crystals containing C, H, O, and N atoms and react by detonation. Propellants and pyrotechnics are generally composites of oxide and fuel that undergo combustion reactions. Many studies are being conducted on increasing the energy and controlling the reactivity of EMs. Decreasing the feature size of constituents down to the nanoscale can reduce the crystal defects of explosives, making them safer, and shortening the mass and heat transfer length of the composites, resulting in dramatic deflagration. The formulations can be optimized through the doping of catalysts and the addition of desensitizers, high-energy additives or agents; such an optimization enables the tailoring of the sensitivity and reactivity by changing the reaction process. In addition to these efforts, preparing EMs with diversiform microstructures by advanced methods, such as sol-gel, spraying, high-energy ball milling, surface decorating, vapor deposition and self-assembly, is also efficient. When ultrasonically mixed, the prepared composite energetic materials or hybrid energetic materials are usually in a state of disunity with a random distribution of components caused by unequal sedimentation velocity in solvents, and unwanted aggregation occurs during drying. The inhomogeneity of EMs leads to poor reactivity over a long diffusion distance and low combustion efficiency. In contrast, the ingenious microstructures, including core–shell, laminate, microcapsule and porous microstructures [1], can provide more reaction channels with a larger contact area and shorter diffusion distances. However, these preparation methods have some limitations in terms of cost, production cycle, scalability, size distribution, and operating conditions [2].

Comparatively speaking, electrohydrodynamic atomization (EHDA) is a more appropriate choice and has been widely applied to micropropulsion, ionization-mass spectrometry, particle deposition, fiber production, film coating, and 3D printing for over one hundred years. Nevertheless, energetic materials have only been processed by EHDA for one decade. At the end of the 2010s, nanofibers of nitrocellulose (NC) were first prepared by electrospinning [3], while nanocrystals of hexogen (RDX) were successfully produced by electrospray crystallization [4]. Then, efforts to control the reactivity and output performance of EMs with designed microstructures by EHDA became active, and a considerable number of academic contributions have been made by researchers worldwide with multidimensional impacts.

Although many systematic reviews have been written on advanced strategies for preparing energetic materials [1,2,5], and the applications of electrospray and electrospinning [6,7,8,9,10,11,12], the combination of EMs and EHDA was only briefly mentioned in a few reviews [13,14,15]. Herein, we mainly focus on the scientific progress of the application of EHDA in the field of energetic materials over the past decade. Firstly, we describe the principle of the EHDA process, including the setup, typical dynamic behaviors and transition between electrospinning and electrospray. Then, we discuss the production of energetic crystals and assembled particles (0D), fibers (1D) and films (2D) with superior reactivity. Finally, we discuss potential 3D structures and development challenges that impede this field from moving out of the laboratory and into practical applications.

## 2. Principles of Electrohydrodynamic Atomization

**Figure 1:**
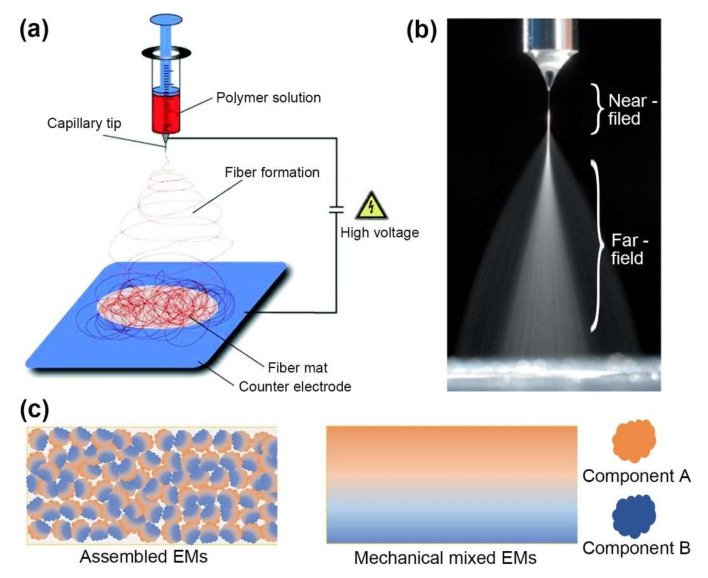
(**a**) Schematic of electrospinning setup. Reprinted with permission from Ref. [23]. Copyright 2007 Copyright John Wiley and Sons. (**b**) Illustration of electrospray process consisting of the Tylor cone, emitted jet and broken-up droplets. Adapted with permission from Ref. [24]. Copyright 2018 Copyright Elsevier. (**c**) Schematic comparison of homogenous dispersed composite EMs (components A and B) assembled by EHDA and heterogeneous dispersed EMs prepared by mechanical mixing.

Electrohydrodynamic atomization, which comprises electrospray and electrospinning, is a physical process induced by an external electric field. The setup of EHDA is relatively simple, consisting of a high-voltage source (positive or negative) that provides external electrical energy and a precursor feeding source mounted with a metal capillary nozzle facing a conductive collector, as shown in Figure 1a. Although EHDA can be operated in an atmospheric environment, the setup is usually placed in a fume hood with good electrical isolation and ventilation to ensure safety while maintaining the temperature and relative humidity of the workspace.

### 2.1. Process of Electrohydrodynamic Atomization

When the precursor flows through the metal capillary nozzle, the competition of the external electric force and surface tension deforms the meniscus. At a low voltage, the external electric force is too weak to overcome the surface tension, and then the precursor flows dropwise, which is known as the dripping mode. When the applied voltage is increased, a stronger external electrical force causes the meniscus to elongate into a cone shape known as the Taylor cone. Then, a fine jet (much finer than the nozzle) is ejected from the tip of the Taylor cone; this phenomenon is called the cone-jet mode and is often used due to its repeatability and controllability.

#### 2.1.1. Electrospray

When Newtonian liquids or dilute viscoelastic liquids are used, the emitted jet breaks into fine, charged droplets due to the instability caused by the accumulated surface charge, termed electrospray, as shown in Figure 1b. The diameter of the charged droplets (*d_d_*) can be estimated by Equation (1) [16]:

(1) $$d_{d}=\alpha {\left(\frac{Q^{3}\varepsilon_{0}\rho }{\pi^{4}\sigma \gamma }\right)}^{1/6}$$

where *α* is a constant, *Q* is the solution flow rate, *ρ* is the solution density, *γ* is the solution conductivity, *σ* is the liquid surface tension, and *ε_0_* is the electric permittivity of the free space. Then, these charged droplets fly to the collector, driven by electrostatic force and accompanied by solvent evaporation. Due to solvent evaporation, the droplets shrink, and then the surface charge density increases. When the surface charges of the droplets accumulate and reach the Rayleigh limit, electrostatic repulsion and Coulomb fission occur, forming smaller droplets. This threshold of surface charge *q_R_* can be estimated by following equation [17]:

(2) $$q_{R}=2\pi {\left(16\sigma \varepsilon_{0}R^{3}\right)}^{1/2}$$

where *R* is the droplet radius. If the solvent in the charged droplets completely evaporates before landing on the substrate, then dry particles with a narrow size distribution can be collected. After evaporation, the remained diameter (*d_p_*) of solid particles can be deduced by mass conservation [16,18]

(3) $$d_{p}=d_{d}{\left(\frac{\omega \rho_{s}}{\omega \rho_{s}+\left(1-\omega \right)\rho_{p}}\right)}^{1/6}$$

where *ω* is the weight fraction of solid material, *ρ_s_* is the density of the solvent and *ρ_p_* is the density of solid material. When solvent evaporation is insufficient, wet or semidry particles coat the surface of the collector, forming a thin film. The drying degree of the deposited particles can be tuned by choosing solvents with different saturated vapor pressures.

#### 2.1.2. Electrospinning

When viscoelastic precursors with dissolved polymers are used, the emitted jet experiences bending instability driven by an external electrical field and then elongates into filament due to enough polymer chain entanglements; this process is known as electrospinning. Based on volume conservation, the diameter of electrospun fibers *d_j_* can be predicted by the following equation [19] without considerations of elastic effects and solvent evaporation:

(4) $$d_{j}={\left(\frac{2\sigma \varepsilon Q^{2}}{\pi I^{2}\left(2\ln\chi -3\right)}\right)}^{1/3}$$

where, *ε* is the dielectric constant of the outside medium, *I* is the electric current through the jet, and *χ* is the dimensionless wavelength of the bending instability. Then, solidified fibers with diameters down to the nanoscale deposit on the collector and form a nonwoven mat.

### 2.2. Transition between Electrospinning and Electrospray

As shown in Figure 1a,b, the basic principles and setups of electrospray and electrospinning are similar, the only difference being the travel motion of the emitted jet in the external electrical field. Upon the addition of polymers into the precursors, a transition between electrospinning and electrospray may occur, dominated by the various rheological properties of the precursor, such as viscosity, surface tension and chain entanglement behaviors, which are mainly determined by the molecular weight (*M_w_*) and concentration of the polymer.

The precursors can be classified into the dilute regime, the semidilute unentangled regime, the semidilute entangled regime, and the concentrated regime in terms of their polymer concentrations [20]. The jets emitted under these regimes show distinct dynamic behaviors and produce different structures of particles, beaded fibers and uniform fibers under identical operation parameters. In the dilute regime, no overlap of polymer chains occurs. In the semidilute unentangled regime, some polymer chains overlap, but they do not overlap enough to entangle each other. Then, without topological constraints or chain entanglements, the emitted jet breaks into droplets induced by external electrical force and surface tension, namely electrospray. In the semidilute entangled regime, obvious polymer chain entanglements dominate the deformation of the emitted jet, and discontinuous beaded fibers can be produced, in which short nanofibers connect large particles. As the concentration increases, the shape of the beads changes from spherical to elongated and then spindle like, ultimately disappearing, forming continuous fibers in the concentrated regime. The threshold between these regimes can be expressed as the critical chain overlap concentration *c_o_* (boundary of the dilute regime and the semidilute unentangled regime) and the critical entanglement concentration *c_e_* (boundary of the semidilute unentangled regime and the semidilute entangled regime), which can be measured by exploring the dependence of the specific viscosity on the concentration [21].

The molecular weight (*M_w_*) decides the chain length and occupied hydrodynamic volume of the applied polymer and further affects the rheological behaviors and chain overlap behaviors. Polymers with higher *M_w_* possess a higher hydrodynamic volume and consequently have a lower *c_o_* and *c_e_*. For poly(methyl methacrylate) (PMMA) in N,N-dimethylformamide (DMF), the values of *c_o_* are 10.2 wt% and 2.5 wt% when the *M_w_* values are 12,470 g·mol^−1^ and 205,800 g·mol^−1^, respectively [21].

### 2.3. Morphology

As expressed by the aforementioned scaling laws, the size distribution and morphology of the prepared materials can be easily regulated by various parameters, which can be classified as precursor parameters (electrical conductivity, surface tension, viscosity, volatility, concentration, and the molecular weight of polymer), and operation parameters (electric field strength, flow rate, and distance from the needle to collector) [7,16,22]. In addition, ambient parameters [23] (temperature, and relative humidity) may affect the evaporation kinetics and viscosity of precursor, and afterwards influence the process of EHDA. Then, bulk materials can be processed into particles, fibers, films and 3D structures in a “bottom-to-up” manner. Unlike the ultrasonic mixing of composites occurring in a macroscopic system, EHDA is a physical recrystallization or mixing process which may involves a chemical reaction in confined tiny droplets or elongated jets. A benefit of this procedure is that tiny crystals and composite materials with homogeneously dispersed components can be obtained, as illustrated in Figure 1c. The feature size of the obtained materials usually falls within the micron to nanometer range with monodispersity. Fast solvent evaporation leads to pore formation on the surface and the interior of the assembled particles as insufficient time to rearrange polymer chains or solutes, resulting in a porous morphology. In contrast, solvents with a low vapor pressure lead to the formation of smooth morphology. Moreover, particles and fibers with core–shell structures or coaxial structures can be readily obtained by a coaxial needle.

Electrosprayed dry particles are generally wrinkled, hollow spheres, sponge-like spheres or compact spheres; their morphologies are determined by solvent evaporation, the rigidity of the elastic shell, the mechanical balance of inner and ambient pressures, surface tension stress and electrical normal stresses [22]. In the case of film deposition, the electrosprayed droplets must remain wet when landing on the substrate so that the polymer chains or solutes can rearrange before solidification. Therefore, the prepared films are generally dense and have no obvious cracks without further thermal treatment [11]. The electrospun nonwoven mats are composed of fibers with inter-/intrafibrous porosity. After elongation, the diameter of electrospun fibers can be reduced to a few tens of nanometers. With a small diameter, the macropores between the fibers and the mesopores or micropores on the surface of the fibers also contribute to a high specific surface area. This unique structure gives the nonwoven textile specific functionalities, such as filtration, cell seeding and attachment, substance transport and exchange. With these excellent morphologies and structures, EMs can show superior reactivity in the forms of particles (0D), fabric mats composed of nanofibers (1D) and films (2D).

## 3. Particles (0D EMs)

### 3.1. Recrystallization and Cocrystallization of Organic High Explosives

**Figure 2:**
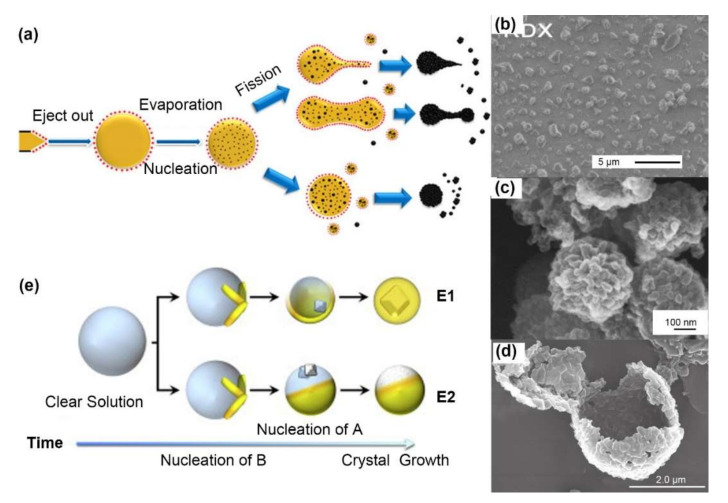
(**a**) Schematic of electrospray formation mechanism of sub-microspheres. Adapted with permission from Ref. [26]. Copyright 2018 Copyright John Wiley and Sons. SEM images of (**b**) electrosprayed RDX nanoparticles from acetone. Adapted with permission from Ref. [25]. Copyright 2014 Copyright Elsevier, (**c**) electrosprayed LLM-105 sub-microspheres stacked by ~50 nm nanoparticles. Adapted with permission from Ref. [26]. Copyright 2018 Copyright John Wiley and Sons, and (**d**) electrosprayed CL-20 hollow microsphere consisting of numbers nanoparticles from ethyl acetate [27]. (**e**) Possible mechanisms in electrospray cocrystallization: heterogeneously nucleation leads to encapsulation (mechanism E1), while low affinity leads to no real encapsulation (mechanism E2). Reprinted with permission from Ref. [25]. Copyright 2014 Copyright Elsevier.

For crystalline explosives, such as nitrate-, nitramine- and nitro-explosives, crystal quality are vital to the stability and sensitivity. Smaller crystal size, less crystal defects (dislocations, inclusions or defects). Recrystallization induced by electrospray is an attractive approach to improving the product quality, which benefits from ultrafast solvent evaporation, as shown in Figure 2a. Electrospray crystallization starts with nucleation on the surface of tiny droplets and then inward extension [25]. Unlike during the cooling crystallization occurring in bulk solvents, which does not encounter any boundaries, the crystal growth occurring during electrospray crystallization is restricted to tiny droplets that evaporate quickly, leading to insufficient time and space for crystal growth with different morphologies. During cooling crystallization, needle-like TNT crystals with a characteristic size of ~200 μm and compact RDX crystals of ~100 μm can be obtained. During electrospray crystallization, spherical TNT crystals and compact RDX crystals with diameters of 1 μm can be produced.

Nanoparticles of RDX, HMX, LLM-105 and CL-20 have been fabricated by this procedure, as summarized in Table 1. Radacsi [4] fabricated submicron single crystals of RDX (~400 nm) with lower impact sensitivity (10 J) and friction sensitivity (>360 N) by electrospray, as shown in Figure 2b. However, when the applied voltage was decreased to 4.5 kV or lower, a certain content of hollow spheres (~4 μm) composed of multiple crystals appeared due to the lower surface charge and less Coulomb fission. Similar structures, 200~400 nm spheres of LLM-105 [26] composed of ~50 nm particles (Figure 2c) and microsized hollow spheres of CL-20 [27] aggregated by smaller particles, were also observed, as shown in Figure 2d. When the ethyl acetate was replaced with acetone, single crystals of CL-20 only ~180 nm long were produced. The unique complex structure composed of multiple crystals can be maintained for at least 6 months under atmosphere, without obvious particle growth induced by Ostwald ripening or aggregation [26].

The calculated crystal growth rate in the electrospray crystallization process within an ultrashort time (~1.08 ms) is nearly 6000 times higher than that of cooling crystallization [4]. Therefore, the crystal phase of the obtained particles may be different from that of the raw materials due to insufficient crystal growth. Peaks with lower intensities and broader widths were found in the X-ray diffraction (XRD) pattern of electrosprayed LLM-105, implying the presence of crystal imperfections; these peaks were not observed in the pattern of raw LLM-105. The β-phase of electrosprayed CL-20 [27,28], preferentially formed because it had the lowest lattice energy, and there was not enough time for it to transform into the ε-phase, which is the most strong and stable phase.

Building cocrystalline explosives is an efficient way to increase the energy density and improve the detonation performance with increased safety over that of the starting materials. Reus [25] investigated the concomitant crystallization of TNT and RDX by an electrospray system. Potential crystallization mechanisms were proposed, as shown in Figure 2e. Heterogeneous nucleation led to encapsulation (mechanism E1), while low affinity prevented encapsulation (mechanism E2). The prepared TNT/RDX powders were less sensitive, but they had a higher impact (15 N·m) and greater friction sensitivity (>360 N) than those of the starting RDX (4 N·m and 120 N). CL-20-based cocrystals, including partial cocrystals of CL-20/TNT, pure cocrystals of CL-20/TNB, and possible cocrystals CL-20/DNB, were prepared and verified by PXRD, FTIR and terahertz (THz) spectroscopy [28].

**Table 1:** Summary of organic explosives recrystallized and cocrystallized by electrospray.

Authors	Energetic Materials	Solvent	Operation Parameters Needle Diameter; Flow Rate; Distance; Applied Voltage	Feature
Radacsi [4]	RDX 20.8 mg/mL	DMK	0.15~0.58 mm; 1~5 mL/h; 10~35 cm; 3.8~4.8 kV	200 nm~600 nm
Radacsi [29]	RDX HMX			200~600 nm RDX spheres; 200~500 nm HMX spheres; 1 μm HMX donut particles
Reus [25]	TNT 42~840 mg/mL RDX 54~60 mg/mL	DMK	0.61 mm; 0.5~1.5 mL/h; 3~7 cm; −3.5~−7 kV	submicron RDX (core)/TNT (shell)
Huang [26]	LLM-105 0.7%wt	DMF+ NMP (*v*/*v* = 6/1)	19G~27G; 0.025~0.075 mm/min; 25 cm; −15 kV to 7~9 kV.	200~500 nm spheres stacked with 50 nm nanoparticles
Huang [28]	CL-20/TNT, CL-20/DNB CL-20/TNB 100 mg/mL	DMK; EAC; MEK; BAC	27G; 0.05 mm/min; 20 cm; 5~8 kV to −10 kV.	1~2 μm CL-20/TNT partial cocrystal, 100~500 nm CL-20/DNB cocrystal, 200~600 nm CL-20/TNB cocrystal
Yan [27]	CL-20 20 mg/mL	EAC; DMK	0.21~0.86 mm; /; 5–12 cm; 4~8 kV;	~2.8 μm hollow sphere (ethyl acetate); 320~610 nm nanoparticles (acetone)

### 3.2. Assembled Particles of Composite Energetic Materials

**Figure 3:**
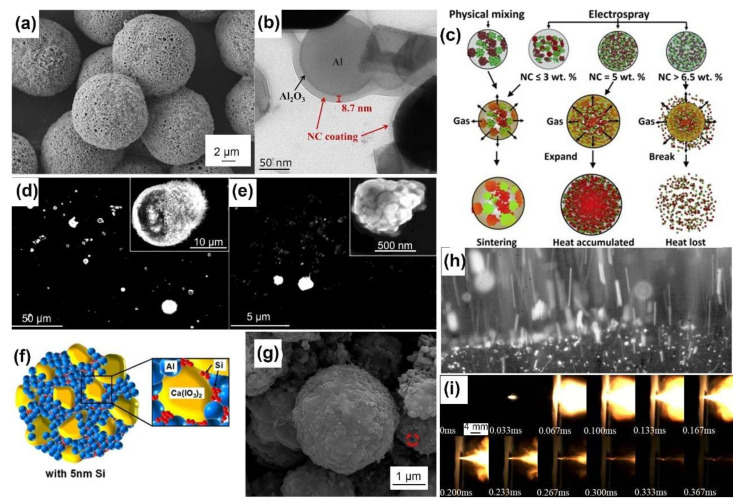
(**a**) SEM image of Al/CL-20 (30 wt%)/NC (2.5 wt%) microspheres prepared by electrospray. Adapted with permission from Ref. [49]. Copyright 2020 Copyright Elsevier. (**b**) TEM image of electrospray assembled Al/Bi_2_O_3_/NC particles, of which nanoparticles were coated by the NC matrix. Adapted with permission from Ref. [50]. Copyright 2018 Copyright Elsevier. (**c**) Proposed mechanism of the sintering prohibition of nano-Al (red) by gaseous products of NC (light blue). Reprinted with permission from Ref. [34]. Copyright 2014 Copyright Elsevier. SEM images of combustion products of (**d**) commercial nano-Al and (**e**) electrosprayed Al/NC mesoparticles with high magnification insets. Adapted with permission from Ref. [35]. Copyright 2016 Copyright Elsevier. (**f**) Schematic illustrating the filling of nSi into Al/Ca(IO_3_)_2_ mesoparticle voids. Adapted with permission from Ref. [40]. Copyright 2020 Copyright American Chemical Society. (**g**) SEM image of electrosprayed Al/CuO/PVDF/RDX(30 wt%) microspheres without distinct void on the surface. Adapted with permission from Ref. [41]. Copyright 2020 Copyright Elsevier. (**h**) Snapshot of AP/HTPB/Al propellant (Al/NC mesoparticle based) burning at atmospheric pressure. Adapted with permission from Ref. [44]. Copyright 2015 Copyright John Wiley and Sons. (**i**) Combustion of electrosprayed Al/CuO/NC mesoparitcles loaded in a microthruster. Reprinted with permission from Ref. [46]. Copyright 2017 Copyright Springer.

Composite energetic materials, such as propellants and pyrotechnics, are composed primarily of fuels and oxides, and they sometimes contain additional functional additives. In general, Al, B, and Si are the most studied fuels. The oxidization of metallic oxides, inorganic salts and fluorine-containing substances is being explored. The redox process of composite energetic materials is determined by the heat transfer and mass transport among particles. Through electrospray, heterogeneous components can be assembled with intimate contact, homogenous dispersion, and enhanced reactivity and energy release, as summarized in Table 2. The assembled particles with narrow size distributions are generally highly spherical, nearly spherical, or irregular in shape. They have porous surfaces with numerous pores on the scale of tens of nanometers, as shown in Figure 3a; these pores form because from the unequal solvent evaporation rates at the surface and interior of the tiny droplets. The pores can provide additional transport channels for the diffusion of gaseous products and heat transfer via convection. Insoluble solid particles and recrystallized components are dispersed evenly with intimate interfacial contact and are encapsulated in the matrix of the polymer (or the core–shell microsrtructure) [30,31], which can be clearly seen from transmission electron microscopy (TEM) images, as shown in Figure 3b.

Under a high heating rate, nanoparticles of aluminum (nano-Al) suffer from ultrafast and serious coalescence and then lose nanostructures due to reduced surface energy within 50 ns; this phenomenon may be the main reason why the reactivity is lower than expected value [32]. To solve this problem, Wang [33] assembled gas agent of NC and nano-Al into meso-particles by electrospray. During combustion, the assembled mesoparticles were broken into isolate burning Al nanoparticles in situ by hot gaseous products of NC, preventing sintering while providing an oxidizing environment, resulting in more efficient utilization of the nanostructure, as shown in Figure 3c [34]. However, the physically mixed powders exhibited a much darker flame and suffered from severe sintering. Jacob [35] characterized the combustion performance of Al/NC mesoparticles in a Hencken burner, exhibiting at least an order of magnitude lower average burn time with a much narrower flame shape than that of nano-Al. The SEM images of quenched post-combustion particles clearly show that the products of the assembled mesoparticles were smaller than those of the pristine powder, as shown in Figure 3d,e [35], verifying that electrospray is an effective way to retain the nanofeatures of the components. Similar to nanoparticles of Al, nano-B and nano-Si have also been processed by electrospray to address the low combustion efficiency and difficult ignition. Cheng [36] decorated boron nanoparticles (nano-B) with nano-Al and PVDF by electrospray. Within the assembled microspheres, PVDF could consume the oxide shell of nano-B to remove the reaction barrier, while the reaction between nano-Al and PVDF promoted the second oxidation reaction of nano-B. Zuo [37] maximized the heat release by encapsulating nano-Si and ammonium perchlorate (AP) into the NC matrix, which facilitated the elimination of the thin silica shell driven by perchloric acid.

Other polymers have also been applied. Yang [38] applied polyvinylidene fluoride (PVDF) as a binder to promote the reactivity of aluminum nanoparticles. Benefiting from the corrosion of the alumina shell by the products of PVDF decomposition, the combustion duration of Al/PVDF decreased by one order of magnitude (3.51 s vs. 219 ms). Yan [39] exploited the energy function of glycidyl azide polymer (GAP) to nano-Al. The nano-Al/GAP microspheres generated a higher peak pressure but slower pressurization rate than that of the nano-Al/NC microspheres. Huang [28] prepared Al/CL-20 microspheres with F2314 to improve the reactivity of nano-Al. The assembled microspheres exhibited a higher maximum pressure, faster pressurization rate and more violent combustion than the physical mixture.

Pores can improve the reactivity of electrosprayed mesoparticles but lead to poor packing density and energy density. Therefore, filling these voids with energetic components can compensate for the reduced volumetric energy density. To this end, Ghildiyal [40] used 5.0 wt% plasma-synthesized Si nanoparticles (~5 nm) as the void filler of Al/Ca(IO_3_)_2_/PVDF, an energetic-biocidal composite, as shown in Figure 3f. The addition of nano-Si led to a ~21% enhancement in energy density and a 2~3-fold increase in pressurization characteristics, benefiting from the oxygen diffusion kinetics of the nano-Si. Xiao [41] increased the mass content of RDX (30 wt%) to fill the voids of the Al/CuO/PVDF mesoparticles and increase the density of the particles, as shown in Figure 3g.

As the problem of severe nano-Al sintering has been solved, the nanothermites assembled by electrospray exhibit superior reactivity. Monk [42] compared the combustion performance of Al/CuO prepared by different methods of ultrasonic mixing, electrospray and arrested reactive milling (ARM) under an inert atmosphere (argon or helium) and air atmosphere. The results showed that electrosprayed particles generate the highest pressure within the shortest combustion duration and form the finest primary combustion products of Al_2_O_3_ and Cu, implying the best utilization of the thermite reaction. Several attempts at applying these electrosprayed particles have been conducted in labs. The combination of heat, pressure and iodine made the metal iodate-based energetic composites (Al/Bi(IO_3_)_3_/NC, Al/Cu(IO_3_)_2_/NC, and Al/Fe(IO_3_)_3_/NC) develop bactericidal properties and exhibit high sporicidal rates [43]. Young [44] added the assembled mesoparticles of Al/NC into AP/HTPB composite propellants with fewer processing constraints. Unlike pure Al nanoparticles, adding the mesoparticles caused combustion to occur closer to the propellant surface with less surface agglomeration and more heat feedback, as shown in Figure 3h, further increasing the burning rate up by ~35%. We also investigated the possible application for a solid propellant microthruster array [45,46]. Electrosprayed Al/CuO/NC powders combust more violently and efficiently, as shown in Figure 3i, leading to a ~50% increase in the specific impulse compared to the mechanically mixed counterpart, 27.2 s vs. 17.7 s. The addition of 10 wt% ammonium perchlorate (AP) increased the specific impulse of Al/CuO/NC from 22.2 s to 61.0 s [45]. Electrospray-assembled Al/CuSO_4_·5H_2_O particles can replace primary explosives and directly initiate the detonation of the RDX loaded in a detonator [47]. Essentially speaking, an electrospray is an atomization technology used to generate fine droplets for subsequent applications. Mei [48] atomized a Ni(CH_3_COO)_2_·4H_2_O solution for the reactive precipitation synthesis of Ni(IO_3_)_2_·4H_2_O nanoparticles, as they are a promising metal iodate oxide of anti-biological agents.

**Table 2:** Summary of energetic composites prepared by electrospray.

Authors	Energetic Materials	Solvent	Operation Parameters Needle Diameter; Flow Rate; Distance; Applied Voltage	Size
Wang [33]	Al/NC 173 mg/mL	NC 17 mg/mL; EA + DEE (*v*/*v* = 3:1)	coaxial needle 17G/22G; 0.5 mL/h; 10 cm; 19 kV	2~16 μm
Yang [38]	Al/PVDF 100 mg/mL	PVDF 15 mg/mL; DMK/DMF (*v*/*v* = 2:1)	0.51 mm; 3 mL/h; 10 cm; 18 kV	1~5 μm spheres
Yan [39]	Al/NC Al/GAP	NC; GAP 5wt%; DMK + EAC (*v*/*v* = 4:1)	0.8 mm; 0.5~1.0 mL/h; 10~15 cm; 24 kV	1~6 μm
Cheng [36]	Al/B/PVDF 100 mg/mL	PVDF 10 mg/mL; DMK + DMF (*v*/*v* = 5:1)	0.51 mm; 1.5 mL/h; 10 cm; 18 kV	1~5 μm spheres
Wang [31]	Al/AP/NC	NC DMK + MT+ EA+ DEE (*v*/*v* = 2:10:3:1)	0.20 mm; 0.2~1.0 mL/h; 2.5 cm; 18 kV	0.2~4 μm
Zuo [37]	AP/Si/NC	NC; DMK + DMF (*v*/*v* = 4:1)	22G; /; 9 cm; 18 kV to −2 kV	~10 μm spheres
Yao [51]	RDX/polymer 14.3~20 mg/mL	PVAc, PVB, F2604, DOS, 0.7~1.0 mg/mL; EAC; DMK	/; 1.0 mL/h; 10 cm; 19 kV	1~4 μm spheres
Han [52]	RDX+CeO 40.8 mg/mL	DMK	/; 4.5 mL/h; 10 cm; 19 kV	2 μm spheres
Wang [33]	Al/CuO/NC 210 mg/mL	NC~21 mg/mL; EA + DEE (*v*/*v* = 3:1)	0.43 mm; 4.5 mL/h; 10 cm; 10 kV to -9 kV	2~16 μm
Zhao [53]	Al/Ti/I_2_O_5_/NC 100 mg/mL	NC 5 mg/mL; EA + DEE (*v*/*v* = 3:1)	/; 2.0 mL/h; 15 cm; 20 kV	5~10 μm
Wang [43]	Al/NC/Bi(IO_3_)_3_; Al/NC/Cu(IO_3_)_2_; Al/NC/Fe(IO_3_)_3_ 116 mg/mL	NC 6 mg/mL; EA+ DEE(*v*/*v* = 19:1)	0.43 mm; 4.5 mL/h; 10 cm; 8 kV	3~5 μm; 2~4 μm; 5~7 μm
Dai [50]	Al/Bi_2_O_3_/NC 133 mg/mL	NC 1.3~13.3 mg/mL; EA + DEE (*v*/*v* = 3.5:1)	/; 3.0 mL/h; 10 cm; 18 kV	
Song [54]	Al/MnO_2_/co(PVDF-HFP)	co(PVDF-HFP); EA+ DMF	0.43 mm; 4.0 mL/h; 10 cm; 14 kV	
Song [55]	Al/MnO_2_ 25 mg/mL Al/MnO_2_/KClO_4_~30 mg/mL	EA + DI (*v*/*v* = 3:1)	0.43 mm; 4.0 mL/h; 15 cm; 13 kV	
Chen [56]	Al/MoO_3_/PVDF	PVDF; DMF + CYH	0.42 mm; 4.0 mL/h; 10 cm; 13.5 kV	evenly distribution of Al/MoO_3_/ PVDF
Mei [57]	Al/Mn(IO_3_)_2_/NC 95 mg/mL	NC 4.5 mg/mL; EA + DEE (*v*/*v* = 3:1)	0.43 mm; 2.0 mL/h; 10 cm; 19 kV	2~4 μm
Yi [47]	Al/CuSO_4_·5H_2_O/NC	NC 4 wt %; IPA	/; 4.5 mL/h; 10 cm; 19 kV	CuSO_4_·5H_2_O(1 μm) covered with nano-Al
Ghildiyal [40]	Al/Si/Ca(IO_3_)_2_/PVDF	PVDF 16.7 mg/mL; DMK + DMF (*v*/*v* = 3:1)	0.43 mm; 2.0 mL/h; 10 cm; 19 kV	3~5 μm
Huang [49]	Al/CL-20/NC; Al/CL-20/F2314 102.5 mg/mL	NC; F2314 2.5 mg/mL; EAC	19G; 0.25 mm/min; 20 cm; 6.5 kV to −10 kV	8~16 μm(NC) 8~18 μm(F2314)
Yan [58]	Al/Viton/RDX	Viton; DMF + EAC (*v*/*v* = 10:3)	coaxial needle 1.45 mm/ 0.57 mm; 0.4~0.5 mL/h; 15 cm; 15.5 kV	450~750 nm hollow spheres
Yan [59]	Al/NC(shell)/RDX(core)	NC 5~15 wt%; DMK + EA; DMK+ EAC	coaxial needle 1.45 mm / 0.57 mm; 1.0 mL/h; 10~15 cm; 12~26 kV	500~2000 nm
Yang [60]	Al/Fe_2_O_3_/RDX/NC 115~125 mg/mL	NC 5.0 mg/mL; DMK	0.8 mm; 3.0 mL/h; 6 cm; 18 kV	
Chen [61]	Al/CuO/NC/CL-20 125 mg/mL	NC 6.3 mg/mL; DMK, EAC, EA + DEE, NPA + DEE	0.43 mm; 1.75 mL/h; 15 cm; 17 kV to −3 kV	3~6 μm clay-like or granular particles
Xiao [41]	Al/CuO/PVDF/RDX, 200 mg/mL	PVDF 10 mg/mL; DMK + DMF(*v*/*v* = 4:1)	23G; 0.14 mm/min; 10 cm; 19 kV	2~4 μm

## 4. Energetic Fibers (1D EMs)

Compared to melt blowing, extrusion, or gel spinning, electrospinning can produce uniform fibers from a wide variety of soluble or fusible polymers with tunable diameters ranging from nanometers to micrometers. These nonwoven mats comprised of nanofibers have been extensively applied in wound healing, biomedical scaffold construction, drug delivery, filtration, energy storage, food packing and catalysis. The superior features of small diameters, high porosities and high specific surface areas dramatically alter the reactivity and mechanical properties of electrospun nanofibers in comparison to those of their bulk counterparts, promoting their applications in propellant reinforcement and flexible actuators.

Precursors only containing solutes with small molecular weights or insoluble particles cannot be directly electrospun due to insufficient chain entanglement and elongation. Thus, energetic fibers based on organic explosives or metal nanofuels must involve a carrier polymer. To maintain the output performance of the energetic fibers, the carrier polymers must provide heat feedback or facilitate redox reactions to some extent. In this respect, NC, known as a propellant, and fluoropolymers with strong oxidizability, such as PVDF, are good choices.

**Figure 4:**
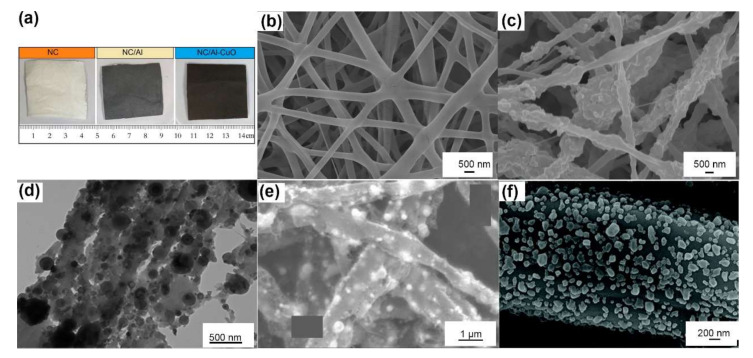
(**a**) Photographs of the as-prepared fibrous mats of NC, NC/Al, NC/Al/CuO (10 cm × 12.5 cm). Reprinted with permission from Ref. [75]. Copyright 2012 Copyright American Chemical Society. SEM images of (**b**) pure NC/GAP fibers, and (**c**) NC/GAP/nano-LLM-105 fibers [62]. (**d**) TEM image of PS fibers with 17 wt% Al/PFPE. Adapted with permission from Ref. [76]. Copyright 2014 Copyright American Chemical Society. SEM images of (**e**) condensed reaction products of PVDF/CuO/Al fibers, Adapted with permission from Ref. [66]. Copyright 2019 Copyright Elsevier, and (**f**) synthesized lead azide particle coating on the surface of carbon fibers. Adapted with permission from Ref. [72]. Copyright 2021 Copyright Royal Society of Chemistry.

Materials prepared by electrospinning are nonwoven mats comprised of fibers, as shown in Figure 4a,b. Energetic fibers based on polymers incorporating high explosives, nanofuels or nanothermites and that have average diameters over the range of 0.1 μm~4.0 μm have been successfully prepared, as summarized in Table 3. The average diameters of fibers are ~2 to 3 orders of magnitude lower than those of the applied needles. The average pore diameters and the specific surface areas of NC/GAP-based nanofibers [62,63] are ~5 nm and ~4.5 m^2^·g^−1^, respectively.

Insoluble nanoparticles or recrystallized solutes are dispersed uniformly on the surface and embedded in the fibrous matrix, as shown in Figure 4c,d. These solid nanoparticles can squeeze into the spaces of the polymers, resulting in incomplete coating and a surface that is rougher and more irregular than the smooth surfaces of pure polymer nanofibers. The addition of solid particles can improve the stress–strain behaviors of a single fiber. Young’s modulus of a single RDX/NC fiber (153 GPa) is ~3 times higher than that of a pure NC fiber (47 GPa), as measured by atomic force microscopy (AFM) [64]. However, excess loading of solid particles leads to severe agglomeration, which damages the mechanical properties. For example, the elastic modulus of NC fibers increased from 71 GPa up to 96 GPa with 5 wt% Fe_2_O_3_ loading and then was reduced to 48 GPa by loading 10 wt% Fe_2_O_3_ [65].

The polymer matrix of nanofibers may act as a protective coating. Lyv [66] found that the uniform coating of PVDF in Al/CuO fibers can increase antioxidation capability with a 57.65% decrease in weight compared to the mechanical mixture. Wang [67] also reported the superior hydrophobicity of GAP/NC/Al@PVDF core–shell nanofibers with a superior reactivity of ~10× higher burning speed compared to Al/PVDF uniaxial fibers; these core–shell fibers can also be excited by a semiconductor bridge in situ. Zhang [68] measured the corrosion resistance of Si/PVDF nanofibers to deionized water and NaOH solution. After 72 h of immersion, the retained content of Si was 100% and 73.3%, respectively, indicating superior storage stability.

Interestingly, after combustion or carbonization, the fibrous mat may maintain its skeleton structure with only carbon residues, as shown in Figure 4e. Therefore, energetic fibers can be prepared by in situ pyrolysis and reaction. Wang [69] prepared Al/Fe_2_O_3_/PVP fibers by the thermal decomposition of Fe(NO_3_)_3_·9H_2_O/Al/PVP fibers. Xie [70] applied CuO fibers originating from CuCl_2_/NC fibers to catalyze ammonium perchlorate decomposition. Wang [71] and Yan [72] have successfully utilized this approach to the in situ synthesis of flexible copper azide (CA) and lead azide (LA) films, respectively, as shown in Figure 4f; these films are compatible with microelectromechanical systems (MEMS). This approach started with electrospinning PAN or PVA fibers, which were then carbonized at 600 °C under a nitrogen atmosphere, followed by a solid–gas azide reaction in situ. The high thermal conductivity and high electrical conductivity make carbon fabric a positive additive for energetic materials. For example, 10 mg of the fabricated CA@C film can directly detonate CL-20 in a micro flyer initiator.

Electrospun fibers can also act as backing materials. He [73] immersed electrospun Al/PVDF fabrics into a precursor of energetic metal organic frameworks (EMOFs) and then placed them in a hydrothermal reactor to obtain a composite mat of Al/PVDF@EMOF, which had a burning rate that was ~5× higher than that of the mechanically mixed composite. The excellent performance can be attributed to the in situ formation of CuO by EMOF thermolysis and higher number of reaction channels on the surface of the Al/PVDF fibers. Li [74] added CL-20 solution dropwise into electrospun polyacrylonitrile (PAN) fibrosa, resulting in a flame that was much lighter than that of pure PAN fibers.

**Table 3:** Summary of energetic fibers fabricated by electrospray.

Authors	Energetic Materials	Binders and Solvents	Operation Parameters Needle Diameter; Flow Rate; Distance; Applied Voltage	Average Fiber Diameter
Xie [77]	Al/NC 6~12 wt%	NC 5~10 wt%; DI + DMK (*wt*/*wt* = 1/10)	0.50 mm; 4.0 mL/h; 20 cm; 28~35 kV	83~98 nm
Xie [70]	CuCl_2_/NC 12 wt%	NC 10 wt%; DI + DMK (*wt*/*wt* = 1/10)	/; /; 20 cm; 25 kV	CuCl_2_/NC 300 nm CuO 100 nm
Xu [64]	RDX/NC 200 mg/mL	NC 100 mg/mL; DMK + DMF (*v*/*v* = 2:1)	0.56 mm; 1.8 mL/h; 25 cm; 27 kV	120 ± 20 nm
Clayton [76]	Al/PFPE/PS	PS 30 wt%; DMF	17G~27G; 0.5~1.25 mL/h; 7.6~10 cm; 12~15 kV	1.1~5.4 μm
Li [78]	B/NC 9 wt%	NC 7.5 wt %; DI + DMK (*wt*/*wt* = 1/20)	0.80 mm; /; /; 20 kV	200~520 nm
Yan [75]	Al/CuO/NC	NC; EA + DEE	0.80 mm; 4.5 mL/h; 6 cm; 18 kV	0.3~1.0 μm
Lyu [66]	Al/CuO/PVDF/GO ~200 mg/mL	PVP 140 mg/mL; DMK + DMF (*v*/*v* = 3:7)	0.60 mm; 0.07 mm/min; 15 cm; 0.65 kV/cm	200 nm~4 μm
Zhang [68]	Si/PVDF 150 mg/mL	PVDF DMK + DMF (*v*/*v* = 1:1)	/; /; 10 cm; 14 kV	200~300 nm
Li [79]	Cu(OCH_3_CO_2_)_2_/Al/ PVP 164 mg/mL	PVP 88 mg/mL DMF + EA (*v*/*v* = 5:1)	0.31 mm; 0.6 mL/h; 18 cm; +13 kV/−3 kV	~150 nm
Li [65]	Al/Fe_2_O_3_/NC	NC: 10 wt%; DMK + DMF (*v*/*v* = 2:1)	0.90 mm; 8.0 mL/h; 20 cm; 25 kV	320 nm
Wang [69]	Fe(NO_3_)_3_·9H_2_O/Al/ PVP 231.5 mg/mL Fe_2_O_3_/Al/PVP 168.4 mg/mL	PVP 105 mg/mL; DMF + IPA (*v*/*v* = 1.3:1)	/; /; 15 cm; 15 kV	~1 μm
Wang [80]	Al/NC/RDX	NC 10% wt; EA + DMK (*v*/*v* = 1:1)	/; 0.02 mm/s; 22 cm; +18 kV/−2 kV	1 μm
Pourmortazavi [81]	Al/Fe_2_O_3_/NC/DAF	NC MEK	0.90 mm; 15.0 mL/h; 10~20 cm; 18 kV	80~232 nm
Luo [62]	NC/GAP/LLM-105 12 wt%	GAP + NC 80.2 mg/mL; DMK	0.80 mm; 3~5 mL/h; 12 cm; 12~18 kV	758 nm.
Luo [82]	NC/GAP/TATB: 12 wt%	NC/GAP 9 wt% DMK	0.80mm; 4.0~6.0 mL/h; 12cm; 12~18kV	1036 nm
Song [63]	F_2602_/GAP/CL-20 20 wt%	F_2602_ + GAP 2~6 wt%; DMK	/; 5 mL/h; 12 cm; 10~20 kV	377~481 nm
Wang [83]	NC/GAP/HNS 12 wt%	NC/GAP 9 wt% DMK	0.80 mm; 3.0~5.0 mL/h; 12 cm; 12~18 kV	1074 nm
Wang [67]	PVDF(shell)/Al/GAP/NC 120 mg/mL(shell)	GAP/NC; DMF/THF	coaxial needle 17G/ 22G; 0.6 mL/h (shell) + 0.06 mL/h (core); 18 cm; +15 kV/−2 kV	578 nm
Yan [72]	Lead Acetate/PVA 20 wt%	PVA 20 wt%; DI + AA (*v*/*v* = 6:1)	0.40 mm; 1.0 mL/h; /; /;	~1 μm
Wang [71]	Cu-MOF(HKUST)/PAN	PAN; DMF	0.60 mm; 1.0 mL/h; /; /;	
Li [74]	PAN	PAN 87 mg/mL; DMF	/; 4.0 mL/h; 15 cm; 25 kV	500 nm

## 5. Energetic Films (2D EMs)

For the application of energetic films, high particle loading relative to the binder/polymer is essential to ensure reactivity. However, the preparation of a homogeneous composite film with high particle loading is difficult because of the high viscosity caused by nanoparticles or the destruction of the film structure [84]. Electrospray deposition can solve this problem with the aid of electrostatic forces. During film deposition, charged droplets are still wet when they land on the collector. Then, the rheological nature of the residual solvent can induce the padding of voids and form crack-free films, as summarized in Table 4.

**Figure 5:**
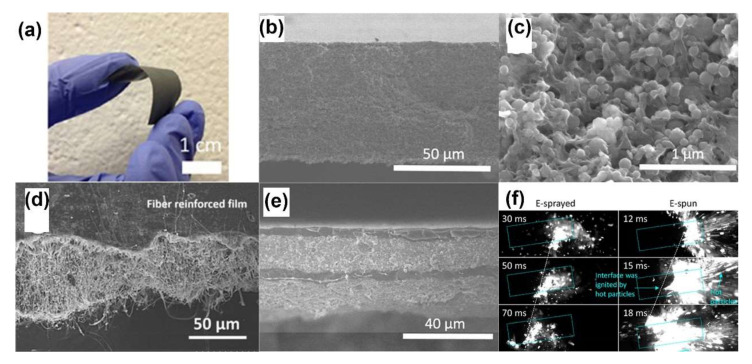
(**a**) Free-standing Al/PVDF film with 50 wt% nanoaluminum loading. Adapted with permission from Ref. [84]. Copyright 2015 Copyright John Wiley and Sons. Cross-sectional SEM images of (**b**,**c**) Al/CuO/PVDF thermite film. Adapted with permission from Ref. [88]. Copyright 2015 Copyright American Chemical Societ, (**d**) fiber reinforced film (average fiber diameter 110 nm). Adapted with permission from Ref. [89]. Copyright 2017 Copyright John Wiley and Sons, and (**e**) four-layer laminate film of Al/CuO/PVDF. Adapted with permission from Ref. [88]. Copyright 2015 Copyright American Chemical Society. (**f**) Burning snapshots of electrosprayed, and electrospun Al/AP/PVDF films. Adapted with permission from Ref. [92]. Copyright 2019 Copyright American Chemical Society.

Using this method, Huang [84] fabricated a flexible, crack-free and free-stand Al/PVDF film with a high solid particle loading of Al up to 50 wt%, as shown in Figure 5a,b. As shown in the SEM image of the enlarged cross-section in Figure 5c, Al nanoparticles were evenly dispersed in the network of the PVDF matrix. DeLisio [85] found that the decomposition of a PVDF matrix can release gaseous HF, which subsequently reacts with the nascent alumina shell of nano-Al in the condensed phase, i.e., preignition reaction (PIR), facilitating the redox reaction of the Al/PVDF films. However, additional pure Al_2_O_3_ weakens the reactivity because the consumption of fluorine leads to the formation of Al_x_O_y_F_z_ species. In addition to being used as propellants, high iodine content energetic films of Al/Bi(IO_3_)_3_/PVDF [86], and multilayers Al/PVDF/I_2_ [87] with excellent combustion characteristics have been fabricated for anti-biocidal applications. After combustion, spores and bacteria can be inactivated and neutralized by the thermal and pressure pulses containing hot gaseous iodine.

Adding nanostructured materials or changing the overall arrangement of layers can enhance the reactivity and mechanical properties of films. Nanofiber-reinforced and laminated multilayered structures have been proposed by Li [88,89] to boost the performance of Al/CuO/PVDF films. PVDF nanofibers were incorporated into the films by combining electrospray and electrospinning with a solid particle loading as high as 70 wt%, as shown in Figure 5d. A finer and higher content of nanofibers can cause the overall equivalence ratio to vary and boost the contact between the reactants, resulting in a higher burn speed. In the laminated film, as shown in Figure 5e, pure PVDF layers were deposited as a supporting layer to maximize particle loading (up to 60 wt% of Al/CuO) and increase mechanical integrity. The laminated film burned fastest when the modulation period decreased to 30 μm for the Al/CuO/PVDF layer and 7 μm for the PVDF layer. It should be noted that PVDF, which had a slower regression rate, could provide an oxidizer to the postreaction region, leading to enhanced global combustion. A contrasting phenomenon reported by Hu [90] is that the polymer matrix of NC combusts firstly due to the low decomposition temperature (~210 °C); then, the loaded particles of AgIO_3_/CB were initiated to a secondary flame, followed by the formation of AgI nanoparticles, which serve as cloud seeding nuclei. Wang [91] employed mesoporous SiO_2_ particles (~0.9 μm diameter), an inert material with low thermal conductivity, as additives to enhance the reactivity of Al/PVDF films. The added particles not only catalyzed the decomposition of Al/PVDF by releasing more HF but also served as embedded ignition points with more thermal feedback to increase the pressurization rates and burning rates by up to 3 times (5 wt% addition). Wang [92] compared the energy release rate (*R_E_*) of Al/PVDF and Al/AP/PVDF films prepared by 3D-direct writing, electrospray and electrospinning, as shown in Figure 5f. Benefiting from the heat feedback of fast-moving hot particles ejected from the burning surface, the electrospun mat exhibited the highest values of *υ* (80 cm/s~100 cm/s) and *T* (1600 K~2200 K), leading to the fastest *R_E_* with an enhancement of 6×~19×. In contrast to direct writing, electrospray and electrospinning have the propensity to promote contact between the reactants and accelerate reactions at lower ignition temperatures.

**Table 4:** Summary of energetic films deposited by electrospray.

Authors	Energetic Materials	Binders and Solvents	Operation Parameters Needle Diameter; Flow Rate; Distance; Applied Voltage	Film Thickness	Combustion Speed
Huang [84]	Al/PVDF 100 mg/mL	PVDF 50~83.3 mg/mL DMF	0.43 mm; 5 cm; 1.5 mL/h; +10 kV (nozzle)/−10 kV (substrate)	170 μm	23 cm/s (air) 11 cm/s (argon)
Li [88]	Al/CuO/PVDF 206.5 mg/mL	PVDF DMF	0.023 mm; 6 cm; 2.0 mL/h; 2~3 kV/cm.	Laminated ~111 μm	16.7 cm/s (argon)
Li [89]	Al/CuO/PVDF (film) PVDF (fiber)	PVDF 7.7~10.4 wt% DMF (film) DMF + DMK (fiber)	0.023 mm; 6 cm (fiber), 10 cm (film); 0.5~1.5 mL/h; 2~3 kV/cm	Fiber reinforced film	~12 cm/s (argon)
Hu [90]	AgIO_3_/CB /NC	NC DMF	0.43 mm; 4.5 cm; 2.0 mL/h; 18 kV	65 μm	4.5 cm/s (air)
Hu [86]	Al/Bi(IO_3_)_3_/ PVDF 113.4mg/mL	PVDF 50 mg/mL DMF	0.43 mm; 4.5 cm; 2.0 mL/h; 18 kV		23 cm/s (air) 5.5 cm/s (argon)
DeLisio [85]	Al/PVDF	PVDF 50 mg/mL DMF	0.43 mm; 4.0 cm; 2.0 mL/h; 18 kV	50~100 μm	5.5 cm/s (argon)
Wang [87]	Al/PVDF+ Al/PVDF/I_2_ 67.4 mg/mL + 404.4 mg/mL	PVDF 50 mg/mL DMF	0.43 mm; 2 cm; 2.0 mL/h; 3.3~5.0 kV/cm	Laminated film 32~124 μm	~35 cm/s (argon)
Wang [91]	Al/PVDF/SiO_2_ ~71 mg/mL	PVDF 50 mg/mL DMF		20~124 μm	~11 cm/s (argon)
Wang [92] (electrospray)	Al/PVDF 152 mg/mL Al/AP/PVDF 193 mg/mL	PVDF 90 mg/mL DMF	/	~600 μm ~310 μm	25 cm/s (air/argon), 5 cm/s (water); 9 cm/s (argon)

## 6. Perspectives

In the last decade, remarkable progress has been made with regard to the successful use of EHDA to prepare energetic materials with advanced microstructures; it is possible to adjust the reactivity and energy release rate. However, there are still some key limitations, and the challenges of further development should be fully addressed to meet practical requirements.

### 6.1. Potential 3D Structures 

Additive manufacturing is an attractive approach to the design and fabrication of structural energetic materials at the micro- and nanoscale with considerable output performance [15]. Unlike traditional mold pressing, casting or slurry curing, 3D printing based on direct writing [93,94] or inkjet printing can directly create complex structures. Although these 3D printing techniques are relatively simple, starting from extruding (or “pushing”) inks onto the substrate and ending with solvent evaporation or polymer curing, they remain limited by poor resolution and high viscosity. Therefore, to improve the resolution and widen the range of viscosity of inks, coupling the EHDA printhead with a moving platform is feasible. Attempts at EHDA printing, a maskless approach with a “pulling” process conducted in microdipping mode or near-field EHDA (electrospray or electrospinning) zone, have been conducted in the fields of electronics and biology.

**Figure 6:**
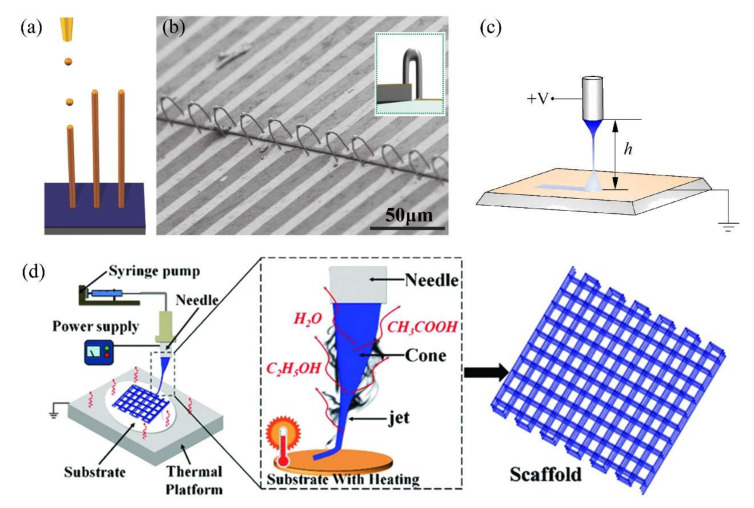
Schematic illustrations of EHDA printing based on (**a**) microdipping (electronic functional ink). Adapted with permission from Ref. [96]. Copyright 2015 Copyright John Wiley and Sons, (**c**) near-field electrospray (zinc oxide lines) [97] and (**d**) near-field electrospinning (biopolymer scaffold). Reprinted with permission from Ref. [98]. Copyright 2018 Copyright John Wiley and Sons. (**b**) SEM image and schematic (insert image) of bridge-like Ag interconnects printed from microdipping mode. Adapted with permission from Ref. [96]. Copyright 2015 Copyright John Wiley and Sons.

In the microdipping mode (Figure 6a), 3D structures can be stacked by drop-on-demand, similar to the ink-jet printing process, but with high resolution, as the droplet size is much smaller than the nozzle [95]. In this way, An [96] successfully printed pillar arrays of Ag, Cu, Co and anthracene, 3D wall structures with high aspect ratios, and freestanding, bridge-like Ag 3D interconnections (1.7 μm diameter × 37 μm length) on flexible substrates (Figure 6b). In near-field electrospray or electrospinning (Figure 6c,d) printing, the distance between the nozzle and substrate is very short (below 10 mm) to prevent the breakup or “whipping” instability of the emitted jet. Thus, a stable, fine jet deposit with a designed route to 3D structures can be obtained with high resolution (the line width is much lower than the inner diameter of the applied nozzle).

However, EHDA printing in the field of energetic materials is still lacking. One fatal flaw is the corona discharge caused by high local electrostatic field strength; this phenomenon occurs when the distance between the nozzle and the substrate is too short. It can also be caused by relatively high local humidity, which is a serious hazard to energetic materials. Another limitation is that EHDA printing can be used to fabricate 3D structures on flat or bent substrates but not within cavities. Therefore, loading energetic materials by EHDA printing into microsystems to form components such as solid propellant microthrusters, microactuators and microinitiators is impracticable because corona discharge may destroy the silicon-based components, and most of the deposited materials would gather on the surface of the cavity rather than at the bottom, forming a layer that could be millimeters thick. Finally, free-standing 3D EM structures without constraints may not combust in the manner of self-propagation. However, integrating microscale 3D energetic materials into actuating systems has not been explored thus far.

### 6.2. Development Challenges

#### 6.2.1. Mass and Continuous Production

The biggest obstacle impeding transfer from research laboratories to commercial applications is the low yield of the product, which has been widely mentioned in previous references [7,13,18]. For example, when the mass loading of energetic materials in the precursor is 200 mg/mL and the feeding rate is 3 mL/h, the mass production rate is only 0.6 g/h without any loss, which is far from practical for applications. To achieve high production, EHDA has been conducted with a capillary array, multiple needles and needleless operation. However, the discontinuous production determined by the hazardous nature of energetic materials and the sedimentation of insolvable solid components should be optimized. Owing to their hazardous nature, the deposited energetic materials must be removed from the collector in a timely manner, then the EHDA process may pause. Otherwise, excessive materials lead to poor conductive then retard the neutralization of redundant charges through the collector, resulting in undesired accidents. The sedimentation of insolvable solid particles may occur over time, which may clog the capillaries and then interrupt the atomization process or produce products with heterogeneous component distributions.

#### 6.2.2. Processing Safety

During EHDA processes, accidental combustion or explosion, high voltage hazard, and the toxicity of the solvents, components and reaction products are potential threats to researchers. The reaction of the deposited energetic materials may be initiated by corona discharge, accumulated charge or other types of stimuli. A high voltage source may output a powerful electric shock. The vapors of applied organic solvents, such as ethyl ether, acetone, acetonitrile, methanol and DMF, are harmful to humans because they can damage the nervous system or respiratory system, and their flammability can lead to an explosion of fuel-air mixtures. The components and reaction products, including organic high explosives (RDX, HMX), heavy metallic substances (CuO, Bi_2_O_3_) and fluorine-containing polymers, are also harmful, particularly their nanoscale counterparts. Hence, valid personal protection should be given more attention.

#### 6.2.3. Binder

As insoluble solid components cannot be processed by EHDA directly, a carrier binder is needed. At present, energetic NC and fluorine-containing PVDF polymers are the most commonly used binders, as they can simultaneously decrease the sensitivity and enhance the outperformance of EMs. The autocatalytic decomposition of NC during storage is an inevitable problem. Fluorinated polymers possess a strong oxidizing capacity; however, their reactivity is much slower than that of nanothermites or hybrid energetic materials. Moreover, good solvents for the fluorinated polymers are restricted by the EHDA requirements, particularly the need for proper volatility and compatibility with other reactants. It is imperative to explore new energetic binders to improve both reactivity and stability.

## 7. Conclusions

Constructing unique microstructures, such as core–shell, laminate, microcapsule and porous microstructures, is an efficient strategy to improve both the high energy output and processing safety of energetic materials (EMs). Electrohydrodynamic atomization (EHDA), which includes electrospray and electrospinning, is a promising strategy that uses an external electric field, enabling it to process bulk materials into particles, fibers, films and three-dimensional (3D) structures under identical operation parameters. The only difference is that the dynamic behaviors of the emitted jet affected by the rheological properties of the precursor.

The nanocrystals and cocrystals of high explosives with high crystalline quality and lower mechanical sensitivity can be obtained by electrospray recrystallization at a much faster crystal growth rate. Through electrospray, composite EMs can be assembled into mesoparticles (0D EMs) with intimate contact, short mass diffusion distance and homogenous dispersion. The introduction of a gas agent can prevent nano-Al from undergoing severe sintering during combustion, leading to a more violent combustion reaction and smaller combustion products. Without the disappearance of nanofeatures, these composite materials can be used as additives, bactericides, primary explosives and solid propellants. In general, energetic nanofibers (1D EMs) consist of carrier polymers and energetic components (high explosives, nanofuels or nanothermites). Insoluble nanoparticles or recrystallized solutes are uniformly dispersed in the fibrous matrix, which can function as a protective coating to prevent unwanted fuel loss in harsh environments. After the carbonization of the pure polymer, the remaining fibrous skeleton structure is beneficial for preparing sensitive EMs with enhanced performance by in situ pyrolysis or dipping. Crack-free energetic films (2D EMs) with high solid particle loadings can be prepared by electrospray with low-volatility solvents, disregarding the high viscosity. Novel structures of nanofiber reinforcement or multilayer (laminated) structures have been proposed to enhance the reactivity and mechanical properties of films, and they can be applied to anti-biocidal, solid rocket, and weather modification applications. EHDA printing can create complex 3D structures with high resolution from highly viscous solutions. However, corona discharge, flat substrate restriction and complex integration processes block the feasibility of printing 3D EM structures by EHDA in the microdipping mode or near-field zone. Before the preparation of EMs by EHDA can shift from the laboratory to practical applications, some key issues, such as scale-up production, personal protection and exploring powerful binders, must be addressed.

## Data Availability

Not applicable.

## Abbreviations

AA	acetic acid
AP	ammonium perchlorate
BAC	n-butyl acetate
CYH	cyclohexane
CL-20	2,4,6,8,10,12-hexanitro-2,4,6,8,10,12-hexaazaisowurtzitane
DAF	3,4-diaminofurazan
DEE	diethyl ether
DI	distilled water
DMF	N, N-dimethylformamide
DMK	acetone
DNB	1,3-dinitrobenzene
DOC	dioctyl sebacate
EA	ethanol
EAC	ethyl acetate
F2314	one of fluoropolymer
F2602	one of fluororubber
F2604	copolymer of vinylidene fluoride and hexafluropropylene
GAP	glycidyl azidepolymer
HMX	octogen
HNS	2,2′,4,4′,6,6′-Hexanitrostilbene
IPA	isopropanol
LLM-105	2,6-diamino-3,5-dinitropyrazine-1-oxide
MEK	methyl ethyl ketone
MT	methanol
MOF	metal–organic framework
NC	nitrocellulose
NMP	N-methyl pyrrolidone
NPA	n-Propyl Alcohol (1-Propanol)
PAN	polyacrylonitrile
PFPE	perfluoropolyether
PS	polystyrene
PTFE/Teflon	polytetrafluoroethylene
PVAc	polyvinyl acetate
PVB	polyvinyl butyral
PVP	polyvinyl pyridine
PVDF	polyvinylidene fluoride
RDX	hexogen
TATB	tramino-trinitrobenzene
TNB	1,3,5-trinitrobenzene
TNT	2,4,6-trinitrotoluene
Viton	dipolymers of hexafluoropropylene and vinylidene fluoride
